# Primary bilateral macronodular adrenal hyperplasia: A rare case report of Cushing syndrome and review of literature

**DOI:** 10.1097/MD.0000000000040050

**Published:** 2024-10-11

**Authors:** Mohammad Reza Ghanbari Boroujeni, Elahe Meftah, Fatemeh Zarimeidani, Rahem Rahmati, Fatemeh Esfahanian

**Affiliations:** a Students Research Committee, Shahrekord University of Medical Sciences, Shahrekord, Iran; b Students’ Scientific Research Center, Tehran University of Medical Sciences, Tehran, Iran; c Department of Endocrinology, Vali-Asr Hospital, Imam Khomeini Hospital Complex, Tehran University of Medical Sciences, Tehran, Iran.

**Keywords:** Adrenal tumor, Cushing’s syndrome, Primary bilateral macronodular adrenal hyperplasia (PBMAH), Unilateral adrenalectomy

## Abstract

**Rationale:**

Primary bilateral macronodular adrenal hyperplasia (PBMAH) is a rare cause of ACTH-independent Cushing syndrome (CS), accounting for <2% of CS cases. Diagnosing PBMAH can be difficult and challenging for clinicians.

**Patient concerns:**

We report a 52-year-old female, a patient with a history of intermittent fever for 3 years. She presented with nausea, headache, and dizziness for several days, along with fatigue, myalgia, muscle weakness, exertional dyspnea, hoarseness, spontaneous bruising over the past several months, and long-term psychological complaints. Additionally, we observed periorbital and facial edema, right lower quadrant tenderness, and abdominal striae during the examination.

**Diagnoses:**

Her laboratory results showed increased cortisol and suppressed ACTH, and an abdominal CT scan revealed 2 heterogeneous masses in the adrenal glands. These findings led us to the diagnosis of PBMAH in this patient. The existence of aberrant receptors was evaluated, and the tests were negative.

**Interventions:**

The patient underwent left adrenalectomy and corticosteroid therapy after the surgery. Her clinical complaints improved after the surgery.

**Outcomes:**

However, her dependency on corticosteroids was not transient after unilateral adrenalectomy, and she still needs glucocorticoid supplementation 1 year after surgery.

**Lessons:**

This patient is a case of PBMAH who presented with fever and CS symptoms and underwent unilateral adrenalectomy. Interestingly, she had suppressed cortisol levels for at least 1 year after the unilateral adrenalectomy. Therefore, we suggest further research on the most effective treatment strategies for PBMAH.

## 1. Introduction

Primary bilateral macronodular adrenal hyperplasia (PBMAH) is an uncommon cause of Cushing syndrome (CS), accounting for <2% of CS cases.^[1–3]^ In PBMAH, enlarged adrenal nodules secrete cortisol independently of corticotropin (ACTH), causing mild to overt CS. PBMAH is often diagnosed incidentally in patients with CS presentation.^[2]^ Three primary pathogeneses have been proposed for PBMAH, including aberrant adrenal expression of ectopic receptors,^[4]^ local ACTH production in the adrenal glands,^[5]^ and genetic mutations such as Armadillo Repeat Containing 5 (ARMC5) mutations.^[6]^ Here, we present a rare case of PBMAH, which experienced intermittent fever for 3 years and was diagnosed with mild CS in the process of the workup for fever of unknown origin (FUO). The patient underwent unilateral adrenalectomy and showed an unexpected hormonal profile during follow-up.

## 2. Case presentation

### 2.1. History

A 52-year-old Caucasian female presented to the emergency department with nausea, headache, dizziness, and right arm pain since the previous days. She reported weakness, lethargy, exertional dyspnea, fatigue, myalgia, spontaneous bruising, bone pain, hoarseness, and productive cough for months. She had experienced a recent unintentional weight gain of 4 kg in about a month and amenorrhea for 5 years. Additionally, the patient had been experiencing depression, drowsiness, irritability, anxiety, nightmares, and restlessness following emotional distress, and she did not comply with the psychiatric treatment. The patient’s past medical history included hypertension for 4 years that was controlled by medication, although she experienced some episodes of elevated blood pressure up to systolic blood pressure of 150 mm Hg.

Additionally, she had grade 1 nonalcoholic fatty liver disease. She had been experiencing intermittent fevers for 3 years, for which she sought a medical consult just before admission, and was under workup for FUO when she was admitted to the ward. She had a history of smoking for 10 years (5 pack-years) and no relevant family medical history. Daily trifluoperazine 1 mg, sertraline 50 mg, nortriptyline 25 mg, and amlodipine 5 mg BID were among her medication history.

The most recent episode of fever and consequent investigations was 3 months before her admission to our center. During these years, procalcitonin, direct Coombs, Brucella agglutination, antinuclear antibody, rheumatoid factor, anti-double-stranded DNA, anti-cyclic citrullinated peptide, Widal test, and Salmonella IgG were evaluated and were all negative.

### 2.2. Investigation

Upon admission, the patient was stable and afebrile, with vital signs recorded as follows: blood pressure of 140/80 mm Hg, pulse rate of 94 beats per minute, respiratory rate of 18 breaths per minute, a temperature of 36.5°C, and oxygen saturation of 95%. Physical examination revealed periorbital and facial edema, commonly referred to as “moon face”, along with right lower quadrant tenderness, abdominal striae, centripetal obesity, and muscle weakness. Initial laboratory assessments of C-reactive protein, liver and thyroid function tests, electrolytes, urea, creatinine, and blood gas were within normal values. However, WBC equal to 18.7 × 10^3^ cells/µL and erythrocyte sedimentation rate of 38 mm/hour were detected in the initial tests (Table 1). The serum antibody screening for Brucellosis, Influenza, Cytomegalovirus, Toxoplasma, Epstein–Barr virus, Herpes simplex virus, and COVID-19 were negative. Considering persistent hoarseness, a laryngoscopy was performed, showing mild vocal cord edema without mass or obstruction.

**Table 1:** Initial laboratory findings after admission.

Variable	Laboratory result	Normal ranges	Variable	Laboratory result	Normal ranges
Hb	**11.9 g/dL**	12–16 g/dL	TSH	0.7 mIU/L	0.4–4.2 mIU/L
WBC	**18.7 × 10^3^ cell/µL**	4–11 × 10^3^ cell/µL	T4	5.8 mcg/dL	4.5–12 mcg/dL
ESR	**38 mm/h**	0–20 mm/h	**T3**	**63.5 ng/dL**	85–205 ng/dL
CRP	**6 mg/L**	0–0.8 mg/L	**TG**	**269 mg/dL**	<150 mg/dL
AST	14 U/L	<35 U/L	**Chol**	**226 mg/dL**	<200 mg/dL
ALT	22 U/L	<35 U/L	**LDL**	**149 mg/dL**	<100 mg/dL
ALP	**268 U/L**	36–150 U/L	Cr	0.6 mg/dL	0.6–1.1 mg/dL
Amylase	37 U/L	<110 U/L	Ca	9 mg/dL	8.5–10.8 mg/dL
Bili (T)	0.3 mg/dL	0.3–1.2 mg/dL	Na	142 mmol/L	135–147 mmol/L
Bili (D)	0.1 mg/dL	0–0.3 mg/dL	K	4 mmol/L	3.5–5.3 mmol/L
PTT	39 s	30–40 s	P	3.3 mg/dL	2.5–4.5 mg/dL
INR	1	0.8–1.2	Mg	2 mg/dL	1.5–2.5 mg/dL
Troponin	**0.018 ng/mL**	<0.01 ng/mL	**HCO**_**3**_	**31.3 mmol/L**	22–29 mmol/L
CPK	37 U/L	10–70 U/L	PH	7.38	7.35–7.45
LDH	**569 U/L**	<240 U/L	**PCO**_**2**_	**53 mm Hg**	35–45 mm Hg
DHEAS	**20 mcg/dL**	35–430 mcg/dL	**ACTH**	**<0.05 pg/mL**	9–52 pg/mL
Metanephrine*	60 mcg/24 h	24–96 mcg/24 h	**Cortisol** †	**16.3 mcg/dL**	5–25 mcg/dL

ACTH = adrenocorticotropic hormone, ALP = alkaline phosphatase, ALT = alanine aminotransferase, AST = aspartate aminotransferase, Bili (D) = direct bilirubin, Bili (T) = total bilirubin, Ca = calcium, Chol = cholesterol, CPK = creatine phosphokinase, Cr = creatinine, CRP = C-reactive protein, DHEAS = dehydroepiandrosterone sulfate, ESR = erythrocyte sedimentation rate, Hb = hemoglobin, HCO_3_ = bicarbonate, INR = international normalized ratio, K = potassium, LDH = lactate dehydrogenase, LDL = low-density lipoprotein, Mg = magnesium, Na = sodium, P = phosphorus, PCO_2_ = partial pressure of carbon dioxide, PTT = partial thromboplastin time, TG = triglyceride, TSH = thyroid-stimulating hormone, WBC = white blood cell.

* Urine content.

† Was done at 7 am.

**Figure 1.**
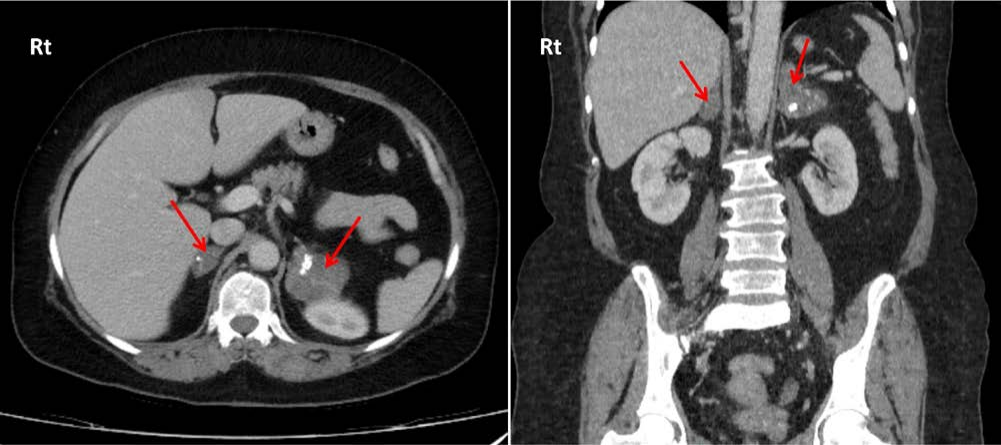
Two heterogeneous masses with small cysts and ring-like calcifications are present in the left and right adrenal glands.

Following psychiatric consultation, the patient was diagnosed with major depressive disorder (MDD) and generalized anxiety disorder (GAD), and her psychiatric medications were changed to gabapentin 100 mg, sertraline 50 mg, and melatonin 3 mg. In the imaging studies, spiral chest computed tomography (CT) scan and abdominopelvic ultrasound were normal. However, an abdominopelvic CT scan revealed 2 heterogeneous masses measuring 48 × 52 mm and 27 × 17 mm in the left and right adrenal glands, respectively (Fig. 1). Both masses had small cysts and ring calcifications with lucent centers resembling phleboliths. There were solid components between the cysts with a density of 31 Hounsfield units (HU) on non-contrast images, increasing to 38 HU in the portal phase and 60 HU in the delayed phase. Despite the presence of cystic and phlebolith-like lymphangioma or venolymphatic malformation, pheochromocytoma could not be ruled out according to the delayed phase enhancement. The smaller right adrenal mass had a density of 7 HU on non-contrast images and was considered insignificant.

We considered these differential diagnoses for the masses: bilateral nonfunctional adrenal adenomas, bilateral pheochromocytomas, bilateral primary hyperaldosteronism, bilateral adrenal metastases, infections (such as tuberculosis, human immunodeficiency virus, etc), and bilateral ACTH-independent cortisol-secreting masses. Next, we conducted laboratory tests for cortisol, ACTH, urinary catecholamine metabolites, and other parameters.

### 2.3. Diagnosis

The urinary catecholamine metabolite tests were negative, excluding pheochromocytoma. On the other hand, the presence of adrenal incidentalomas, along with the level of ACTH (<0.05, reference range 9–52 pg/mL) and cortisol (16.3 after overnight dexamethasone suppression test, reference range 5–25 µg/dL) confirmed an ACTH-independent mild CS coupled with MDD and GAD.

The patient was diagnosed with PBMAH based on the imaging and laboratory findings. We investigated the presence of aberrant receptors by measuring the changes in cortisol and other steroids in response to physiological (upright posture and mixed meals) and pharmacological (metoclopramide and vasopressin) stimuli. The results were negative, indicating that aberrant receptors did not cause hyperplasia in this patient.

### 2.4. Outcome and follow-up

We scheduled to remove the left adrenal gland that had a larger size. The patient fully recovered from the surgery without any complications. The immunohistochemistry and histomorphology results were consistent with the preoperative diagnosis. Her clinical symptoms resolved following the surgery, as indicated and confirmed by multiple post-operative evaluations. Four days postoperatively, the ACTH and cortisol levels were 5.1 pg/mL and 52.44 nmol/L, respectively. In the follow-up 1 month later, laboratory results demonstrated cortisol of 74.20 nmol/L (normal range = 138–690 nmol/L) and ACTH = 11.3 pg/mL, necessitating maintenance of hydrocortisone 20 mg daily. Remarkably, even 1 year after the operation, the ACTH level of the patient was still suppressed (<0.05 pg/mL), and the right adrenal function could not eliminate her need for corticosteroid therapy. However, her symptoms fully resolved, her blood pressure remained controlled, and she expressed well-being in the follow-up outpatient visits.

## 3. Discussion

PBMAH, also known as ACTH-independent macronodular adrenocortical hyperplasia, accounts for <2% of patients with CS.^[3,7]^ The exact prevalence of PBMAH remains unknown, as many cases are incidentally detected on abdominal imaging following the manifestation of CS signs and symptoms. However, as abdominal CT scans become more common, an increasing number of cases are being diagnosed with PBMAH.^[8]^ The mortality and morbidity of PBMAH depend mainly on the treatment received and the complications of CS, such as arterial hypertension and infections.^[9]^ Sporadic PBMAH mainly affects women,^[10]^ while in patients with ARMC5 mutations, it affects both genders equally.^[11]^ It is usually diagnosed in patients aged 40 to 60 years, often after several years or decades of disease progression. Nodule growth and cortisol imbalance appear to progress gradually and without noticeable symptoms.^[12]^ Therefore, the epidemiological data on PBMAH is insufficient, and numerous cases at risk of developing PBMAH and CS complications remain undiagnosed worldwide.

Compared to other adrenal nodules, the nodules found in patients with PBMAH are typically larger than 10 mm in diameter.^[7]^ The largest reported nodule diameter to date is 69 mm.^[13]^ Our patient’s left adrenal gland nodule measures 52 mm, making it one of the largest in nodular adrenal hyperplasia cases. Therefore, it is important to consider the possibility of PBMAH even in cases with larger nodules than expected. Nodule density is also important, typically expected to be <10 HU due to the lipid-rich nature of PBMAH nodules.^[8]^ However, our patient’s nodules exhibited higher density in the portal and delayed phases, indicating that PBMAH nodules can be more solid, and their density may vary depending on their size and duration.

To date, 3 etiological mechanisms have been established to be associated with PBMAH. The most observed one is the aberrant expression of receptors, specifically G-protein-coupled receptors, in the adrenal tissue. Hormones other than ACTH, such as catecholamines, antidiuretic hormone, glucose-dependent insulinotropic polypeptide, thyroid-stimulating hormone, and luteinizing hormone, bind to these aberrant receptors and stimulate cortisol secretion. In a study of 32 cases with CS, at least 1 aberrant cortisol response was detected in 87% of patients.^[14]^ However, the absence of this abnormality in our patient suggests that there could be other underlying causes of adrenal hyperplasia.

The second mechanism is the local production of ACTH in the adrenochromaffin cells of the adrenal glands.^[15]^ This theory challenges the classification of PBMAH as an ACTH-independent disorder, suggesting that it may be considered a pituitary-ACTH-independent cause of CS.

The third mechanism is genetic mutations. Germline mutations of ARMC5, a tumor suppressor gene, have been reported to be the leading cause of familial PBMAH, and the lysine demethylase 1A (KDM1A) gene mutations are responsible for glucose-dependent insulinotropic polypeptide-dependent PBMAH.^[7]^ The family history of our patient was negative for PBMAH and CS; thus, she is possibly a sporadic case. However, genetic testing was unavailable in our center. If feasible, we could explore genetic mutations to enhance our understanding of the patient’s condition.

Cortisol secretion is mildly elevated in PBMAH, and most patients do not exhibit any clinical features. In some cases, PBMAH can result in symptoms of CS, particularly when it progresses to overt CS.^[7]^ If not asymptomatic, patients with PBMAH might exhibit CS signs,^[16]^ similar to our case with CS signs such as moon face, abdominal striae, and muscle weakness. Shah et al^[17]^ reported that a 49-year-old male with PBMAH presented with acute onset of confusion. He was diagnosed with hypercortisolism due to PBMAH and severe psychosis that was unresponsive to haloperidol, lorazepam, risperidone, and citalopram. Our case had MDD and GAD, indicating that a late diagnosis of PBMAH might result in neuropsychiatric complications of CS. We have reviewed the most recent case reports of PBMAH in Table 2.

**Table 2:** Literature review on recent cases with primary bilateral macronodular adrenal hyperplasia.

First author (yr)	Age/sex	Signs and symptoms	Comorbidities	Diagnostic modality	Genetic mutation(s)	Treatment	Outcome
Ghanbari Boroujeni (2024)*	52/F	Nausea, headache, dizziness, arm pain, weakness, lethargy, exertional dyspnea, fatigue, myalgia, spontaneous bruising, bone pain, hoarseness, productive cough, moon face, abdominal striae, centripetal obesity, muscle weakness	Mild CS, FUO, MDD, GAD, hypertension, NAFLD	Lab data, CT scan, and histopathological examination	N/A	Unilateral adrenalectomy	Symptoms improved, but hormonal levels remained abnormal
Wang (2024)^[18]^	41/M	Dry skin, central obesity, multiple ecchymoses, and facial erythema and swelling	Hypertension, pituitary microadenoma and meningioma	Lab data, CT scan, and histopathological examination	A heterozygous mutation, NM_0011052472:c.943C > T (p.Arg315Trp), in ARMC5 gene	Unilateral adrenalectomy	N/A
Tan (2023)^[19]^	66/M	Limping, slender limbs, hypokalemia, dizziness, Buffalo hump, moon face, atrophic skin, and central obesity	Hypertension, epilepsy, chronic hydrocephalus, and systemic rash	Lab data, CT scan, and histopathological examination	ARMC5 gene mutation (chr16:31477527 c..2125 [exon 6] C > T)	Unilateral adrenalectomy	Cryptococcal meningitis after surgery (low-grade fever, fatigue, and low mood and appetite)
Araujo-Castro (2023)^[11]^	64.2 ± 11.2/F: 59.4%	N/A	Hypertension (71.9%), dyslipidemia (56.3%), and T2DM (43.8%)	Lab data and CT scan	N/A	Unilateral adrenalectomy (2 cases) ketoconazole (1 case)	Improvement of cardiovascular risk factors and long-term controlled hypercortisolism
Tang (2023)^[20]^	39/M	Progressive weight gain, severe refractory hypertension, moon face, centripetal obesity, abdominal purple striae, limb edema, and muscle weakness	Severe CS	Clinical, lab data, CT scan, and histopathological examination	ARMC5 germline mutation (c.1855C > T, p. R619*) along with 5 other ARMC5 somatic mutations (four novel mutations)	Unilateral adrenalectomy followed by subsequent contralateral subtotal resection 6 months later	CS symptoms and blood pressure did not improve until subsequent contralateral subtotal adrenalectomy
Fernandez (2022)^[13]^	40/F	Recurrent bilateral blurred vision and weight gain	Severe CS, Resistant hypertension, and T2DM	Lab data, CT scan, and histopathological examination	N/A	Laparoscopic bilateral adrenalectomy	Improved symptoms
Vena (2022)^[21]^	56/M	No sign of CS	Subclinical CS, hypogammaglobulinemia, relapsing human herpes simplex virus infections	Lab data, CT scan, histopathological examination	ARMC5 mutation with a novel somatic frameshift variant in exon 1 (c.231_265del:p.A77Afs*13) and a novel germline variant in exon 6 (c.2436del: p. C813Vfs*104)	Unilateral laparoscopic adrenalectomy	Normalized serum IgG and no more viral infections recurrence
Wang (2022)^[16]^	67/F	Fatigue, edema, moon face, erythematous cheeks, central obesity, thin limbs, atrophic skin, reddish purple striae, ecchymosis, petechiae, and lower extremity edema	Hypertension and intermittent petechiae	Clinical, lab data, CT scan, and histopathological examination	(c.363_373delGCCAGTGCGCC, p. Pro122Alafs*61) in ARMC5 gene	Unilateral laparoscopic adrenalectomy	Improved clinical and lab findings
He (2021)^[22]^	51/M	Uncontrolled hypertension, flushing, plethora, easy bruising, centripetal obesity, supraclavicular fat pad, and buffalo hump	Hyperaldosternism, CS, resistant hypertension, and T2DM	Clinical, lab data, CT scan, and histopathological examination	Germline nonsense mutation of ARMC5 c.967C > T	Bilateral adrenalectomy	Improved symptoms and signs
Nishiyama (2021)^[23]^	64/F	No sign of CS	Subclinical CS, hypertension, dyslipidemia, and T2DM	Lab data, CT scan, and histopathological examination	N/A	Laparoscopic unilateral adrenalectomy	Reduced blood pressure, weight, HbA1C, and insulin requirement
Ferreira (2020)^[12]^	64/M	Atrophic and dry skin, obesity with centripetal fat distribution, multiple ecchymosis, and facial erythrose	T2DM, hypertension, dyslipidemia, and history of MI	Clinical, lab data, CT scan, and histopathological examination	c.1379T < C in the ARMC5 gene	Bilateral adrenalectomy	Improved hypercortisolism
Shah (2019)^[17]^	49/M	Left-sided weakness and confusion, hypercortisolism, hypokalemia, metabolic alkalosis, and venous thromboembolism	Hypertension and severe psychosis	Lab data and CT scan	N/A	Ketoconazole	Improved psychosis, hypertension, and electrolyte abnormalitiesele
Jin (2018)^[24]^	52/F	Progressive bilateral proptosis, proximal muscle weakness, easy bruising, skin atrophy, moon facies, buffalo hump, and abdominal purple striae	CS, hypertension, and T2DM	Clinical, lab data, CT scan, and histopathological examination	ARMC5 germline mutation c.682C > T, along with 5 other somatic mutations	Laparoscopic bilateral adrenalectomy	Improved exophthalmos

CS = Cushing syndrome, F = female, FUO = fever of unknown origin, GAD = generalized anxiety disorder, M = male, MDD = major depressive disorder, NAFLD = nonalcoholic fatty liver disease, T2DM = type 2 diabetes mellitus.

* The present case.

Our patient experienced episodes of fever over 3 years caused by PBMAH, suggesting PBMAH is a possible differential diagnosis of fever. The diagnosis of PBMAH can be challenging due to its rarity and lack of specific signs or symptoms. A recent study suggested using specific criteria to diagnose PBMAH, including clinical manifestation of CS, laboratory tests, macronodules in adrenal CT scans, and pathology results. Genetic studies may also help confirm the diagnosis.^[20]^ Our case fulfilled the mentioned criteria.

Treatment of PBMAH with overt CS typically involves surgery, while medical treatment with adrenal enzyme inhibitors, such as ketoconazole and metyrapone, can be used as a bridge to surgery.^[25]^ Bilateral adrenalectomy was previously utilized in most patients with PBMAH and overt CS, but it leads to adrenal insufficiency and a permanent need for corticosteroid supplementation. More recently, unilateral adrenalectomy has shown promise in successfully normalizing urinary free cortisol levels in patients with less severe CS.^[8,25]^ In contrast to bilateral adrenalectomy, unilateral adrenalectomy mainly results in transient adrenal insufficiency and seems to have less morbidity. However, patients with different severities of CS have been reported to suffer from adrenal insufficiency for months after unilateral adrenalectomy.^[26,27]^ A study focusing on patients with PBMAH with mild to moderate CS highlighted 2 cases with 14 and 60 months of steroid replacement after the procedure.^[28]^ Similarly, our patient still needed exogenous corticosteroids up to the last follow-up 1 year after unilateral adrenalectomy. Overall, the gold standard treatment is still controversial and remains to be determined.^[25]^

## 4. Conclusion

We presented a patient with PBMAH with intermittent fevers who underwent unilateral adrenalectomy. One year after the unilateral adrenalectomy, our patient still has suppressed ACTH and cortisol levels and needs corticosteroid therapy. Further research is necessary to understand the etiology of PBMAH better and to determine the most effective and safe treatment options for patients with this rare adrenal disorder.

## Author contributions

**Conceptualization:** Fatemeh Esfahanian.

**Validation:** Fatemeh Esfahanian.

**Data curation:** Elahe Meftah, Fatemeh Zarimeidani, Rahem Rahmati.

**Investigation:** Fatemeh Esfahanian, Elahe Meftah.

**Resources:** Fatemeh Esfahanian.

**Supervision:** Fatemeh Esfahanian.

**Project administration:** Elahe Meftah, Fatemeh Zarimeidani.

**Writing – original draft:** Mohammad Reza Ghanbari Boroujeni.

**Writing – review & editing:** Rahem Rahmati, Mohammad Reza Ghanbari Boroujeni, Elahe Meftah, Fatemeh Zarimeidani, Fatemeh Esfahanian.
